# The Inhibitory Effect of Somatostatin Receptor Activation on Bee Venom-Evoked Nociceptive Behavior and pCREB Expression in Rats

**DOI:** 10.1155/2014/251785

**Published:** 2014-05-07

**Authors:** Li Li, Rong Luo, Yuan Guo, Fanrong Yao, Dongyuan Cao, Shaojie Ma, Jun Wang, Huisheng Wang, Yan Zhao

**Affiliations:** ^1^Department of Physiology and Pathophysiology, Xi'an Jiaotong University School of Medicine, Xi'an, Shaanxi 710061, China; ^2^Medical Scientific Research Centre, Guangxi Medical University, Nanning, Guangxi 530021, China; ^3^Department of Neural and Pain Sciences, University of Maryland Dental School, 650 West Baltimore Street, Baltimore, MD 21201, USA

## Abstract

The present study examined nociceptive behaviors and the expression of phosphorylated cAMP response element-binding protein (pCREB) in the dorsal horn of the lumbar spinal cord and the dorsal root ganglion (DRG) evoked by bee venom (BV). The effect of intraplantar preapplication of the somatostatin analog octreotide on nociceptive behaviors and pCREB expression was also examined. Subcutaneous injection of BV into the rat unilateral hindpaw pad induced significant spontaneous nociceptive behaviors, primary mechanical allodynia, primary thermal hyperalgesia, and mirror-thermal hyperalgesia, as well as an increase in pCREB expression in the lumbar spinal dorsal horn and DRG. Octreotide pretreatment significantly attenuated the BV-induced lifting/licking response and mechanical allodynia. Local injection of octreotide also significantly reduced pCREB expression in the lumbar spinal dorsal horn and DRG. Furthermore, pretreatment with cyclosomatostatin, a somatostatin receptor antagonist, reversed the octreotide-induced inhibition of the lifting/licking response, mechanical allodynia, and the expression of pCREB. These results suggest that BV can induce nociceptive responses and somatostatin receptors are involved in mediating the antinociception, which provides new evidence for peripheral analgesic action of somatostatin in an inflammatory pain state.

## 1. Introduction

Somatostatin (SST) is a small neuropeptide with wide distribution in the central and peripheral tissues [1–3]. Local administration of somatostatin or its analog octreotide (OCT) reduces lifting/licking behaviors induced by formalin and capsaicin [1, 4] and mechanical hyperalgesia in carrageenan-induced inflammation [5]. In addition, octreotide reduces responses to thermal stimulation in C-mechanoheat sensitive fibers in Sprague-Dawley (SD) rats [1]. However, our previous study indicated that local administration of octreotide did not alter paw withdrawal thermal latency (PWTL) in arthritic Dark-Agouti rats [6]. These inconsistent data led us to further determine the local antinociceptive effects of octreotide.

The bee venom test is a well-established inflammatory pain model. Subcutaneous (s.c.) injection of bee venom solution into a hindpaw of rats and cats has been reported to induce unique expressions of a prolonged, persistent, and spontaneous flinching reflex (lifting and licking behaviors indicative of pain) in a monophasic manner for 1-2 hours followed by a profound, persistent, mechanical, and thermal hyperalgesia in the injured site for 72–96 hours [7–9]. The bee venom (BV) test can closely mimic the complicated state of clinical inflammatory pain caused by tissue injury, which includes both spontaneous nociception and subsequent mechanical and thermal hyperalgesia [7]. The previous studies on the antinociceptive effect of somatostatin or octreotide considered only spontaneous nociception or hypersensitivity [1, 4–6]. Therefore, the inflammatory pain induced by BV and the local antinociceptive effects of octreotide were observed in the present study.

The cAMP response element-binding protein (CREB) is a transcription factor that has been implicated in the transcriptional regulation of many genes [10]. Several studies have shown that the activation of CREB in spinal cord dorsal horn neurons plays a central role in the transmission of nociceptive stimuli [11–13]. CREB phosphorylation at Ser133 has been identified as an essential trigger for CREB activation, which leads to the transcription of a number of immediate early genes, including those coding for the important pain-related proteins c-Fos and cyclooxygenase-2 [10]. An increase of phosphorylated CREB (pCREB) in the dorsal horn of the spinal cord occurs in inflammation pain [14] and neuropathic pain models [15, 16]. After a unilateral injection of formalin into the hindpaw, a strong and bilateral increase of pCREB in the spinal cord was induced [17]. In addition, intrathecal injection of CREB antisense oligonucleotide attenuates tactile allodynia caused by partial sciatic nerve ligation [18]. Thus, pCREB is considered a good marker for neuronal activity after noxious stimulation. Some dorsal root ganglion (DRG) neurons are also pCREB-positive [17]. Accordingly, another aim of the present study was to observe the expression of pCREB induced by BV in the spinal cord and DRG.

## 2. Materials and Methods

### 2.1. Animals

A total of 91 male SD rats weighing 250–300 g were used in the present study (66 for the nociception behavior test and 25 for the immunochemistry experiment). SD rats were provided by the Laboratory Animal Center of Xi'an Jiaotong University School of Medicine. All procedures were approved by the Institutional Animal Ethics Committee of the Xi'an Jiaotong University and were in accordance with the ethical guidelines of the International Association for the Study of Pain. In addition, every attempt was made to minimize the number of animals used. Rats were housed under a 12 h light-dark cycle with food and water available* ad libitum*.

### 2.2. Groups and Pharmacological Intervention

A volume of 50 *μ*L BV (0.1 mg lyophilized whole venom of* Apis mellifera* (Sigma Co., St. Louis, USA) dissolved in 0.9% sterile normal saline (NS)) was intraplantarly injected into right hindpaw to produce nociception [19]. A concentration of 20 *μ*M octreotide diluted in 0.9% NS (Sandostatin, Novartis Pharma Schweiz AG, Bern, Switzerland) was utilized in the present study based on the literature [6].

SD rats were divided into five groups: (1) NS + NS group: 60 *μ*L NS intraplantar injection followed by 50 *μ*L NS injection as the control group; (2) NS + BV group: 60 *μ*L NS intraplantar injection followed by 50 *μ*L BV injection as the pain model group; (3) ipsilateral OCT + BV group: 60 *μ*L octreotide (20 *μ*M) injection prior to the BV injection; (4) contralateral OCT + ipsilateral BV group: 50 *μ*L BV injection into the right hindpaw after injection of 60 *μ*L octreotide into the contralateral hindpaw to determine whether the local injection of octreotide can bring about systemic effects; (5) c-SOM + OCT + BV group: local injection of 60 *μ*L, 128 *μ*M cyclosomatostatin (c-SOM, Sigma Co., St. Louis, USA), a somatostatin receptor (SSTR) antagonist, before the intraplantar injection of 60 *μ*L octreotide to determine whether the effect of octreotide was somatostatin receptor specific. A ten-minute interval was allowed between the applications of each drug in all groups.

### 2.3. Spontaneous Nociceptive Behavioral Observation

Behavioral observations were carried out by the same investigator from 9 to 11 am. All rats were habituated for 3 days before the start of experiment. The rats were placed in a 30 × 30 × 30 cm^3^ transparent glass test box for at least 30 min until their cage exploration and grooming activities ceased. The spontaneous nociceptive behavioral responses were quantified by counting the number of flinches and the cumulative time (in seconds) spent lifting and/or licking the injected hindpaw during each 5 min period for 1 hour after an intraplantar injection of BV [19, 20]. A flinch was defined as a spontaneous, rapid jerk of the foot. Lifting indicated that the injected paw was elevated without touching the floor. Licking meant that the injected paw was licked or bitten.

### 2.4. Measurement of Persistent Mechanical and Thermal Sensitivity

Mechanical sensitivity was calculated by measuring the 50% paw withdrawal mechanical threshold (PWMT) in response to mechanical stimuli [21] as assessed by a set of calibrated von Frey filaments with bending force ranging from 0.4 to 15 g (Stoelting Company, Wood Dale, USA). Beginning with the 2.0 g filament, each filament was applied underneath both hindpaws perpendicular to them with enough force to cause slight buckling for 6–8 s. The pattern of positive and negative responses was converted into a 50% threshold using the formula given by Dixon [22].

Thermal sensitivity was measured as previously described [23]. The animals were placed in a transparent plastic box (28 × 25 × 21 cm^3^) on a 3 mm thick elevated horizontal glass plate. A radiant heat stimulator (BME-410A, Beijing Sunny Instruments Co. Ltd, Beijing, China) was placed under one hindpaw, and the PWTL was determined to an accuracy of 0.01 s. Both hindpaws were tested. A 20 s cut-off was imposed on the stimulus duration to prevent tissue damage. Five stimuli were repeatedly applied to each hindpaw in turn, with a 10 min interval between applications in the same region and 5 min interval between applications in different hindpaws. The PWTL for each hindpaw was defined as the mean value of the latter four stimuli. Based on a previous report [20], the PWMT and PWTL on the injected side and the contralateral hindpaw were investigated 30 min prior to the injection of BV and 2 hours after the injection.

### 2.5. Immunohistochemistry

SD rats were deeply anesthetized with pentobarbital (80 mg/kg, i.p.) and perfused through the left ventricle with 200 mL 37°C warm saline followed by 400 mL of 4% paraformaldehyde in 0.1 M phosphate buffer (PB, pH 7.4, 4°C) 30 min after injection of BV into one hindpaw. The L4-L5 spinal cord segments and L5 DRG were removed, postfixed for 24 hours in the same fixative, and then cryoprotected overnight in 30% sucrose in PB.

Tissues were cut coronally in a cryostat (Leica CM1900, Germany) at 35 *μ*m thickness for the spinal cord and 14 *μ*m for the DRG. One of every three sections through the L4-L5 spinal cord segments and all the L5 DRG sections were collected and rinsed twice in 0.01 M phosphate buffer saline (PBS). The sections were immunostained for pCREB with the avidin-biotin-peroxidase (ABC) method [24]. After being pretreated with 0.3% H_2_O_2_ for 10 min and 5% normal goat serum (NGS) for 1 hour at room temperature, sections were incubated with the primary antibody, anti-pCREB (1 : 100, Cell Signaling Technology, Inc., Beverly, MA, USA), in 5% NGS for 48 hours at 4°C. Subsequently, the sections were incubated overnight with biotinylated goat anti-rabbit IgG at 4°C and further processed using avidin biotin peroxidase complex (ABC, Zhongshan Biotechnology CO., LTD, Beijing, China) according to the instructions of the manufacturer. Between each step, the tissue was rinsed at least 3 times in 0.01 M PBS containing 0.3% Triton X-100 for at least 10 min each. Then, the sections were incubated with 0.02% 3,3′-diaminobenzidine (DAB, Zhongshan Biotechnology CO., LTD, Beijing, China) for 5–10 min. Finally, the sections were mounted onto gelatin-coated glass slides and allowed to air dry. To observe whether pCREB was located in neurons of the DRG, the sections were restained by Nissl's staining method. All sections were then dehydrated through a graded alcohol series, cleaned with dimethylbenzene, and cover-slipped with neutral balsam.

All sections were observed under a light microscope (BX-60; Olympus, Tokyo, Japan). Images were captured using a SensiCam digital camera and imported into SigmaScan Pro Image Analysis Software (SPOT-Insight QE, Diagnostic Instruments Inc., Sterling Heights, MI, USA). To discriminate positive immunostaining from the background, the cells showing a staining twice as intense as the average background were considered positive for pCREB immunoreactivity. In the control experiments, the primary antibodies were replaced with 5% NGS; no positive staining for the replaced antibodies was detected. Four to five random sections from the L4-L5 spinal cord and the L5 DRG were counted and averaged for each animal, and five animals were included in each group [25–27]. Every positive cell was counted in lamina I–VI in the spinal cord. The percentage of neurons stained with pCREB in the DRG was determined by counting the total number of positive neuronal profiles and the total number of neuronal profiles in each section.

### 2.6. Statistical Analysis

All data are presented as the means ± SEM. One-way ANOVA tests followed by the Student-Newman-Keuls method were used to compare differences between the five treatment groups. Paired* t*-tests were used to compare the differences in PWMT and PWTL between pre- and postinjection of BV in the same hindpaw. All analyses were performed with SigmaStat 2.0 software. *P* < 0.05 was considered to be statistically significant.

## 3. Results

### 3.1. Effects of Octreotide on Spontaneous Nociceptive Behaviors Induced by BV

Intraplantar injection of BV produced a prompt, tonic nociceptive response characterized by flinches and continuously lifting/licking of the injected hindpaw for approximately 1 hour. In the control group, there were few flinches observed in the first 5 min, and no lifting/licking occurred.

**Figure 1:**
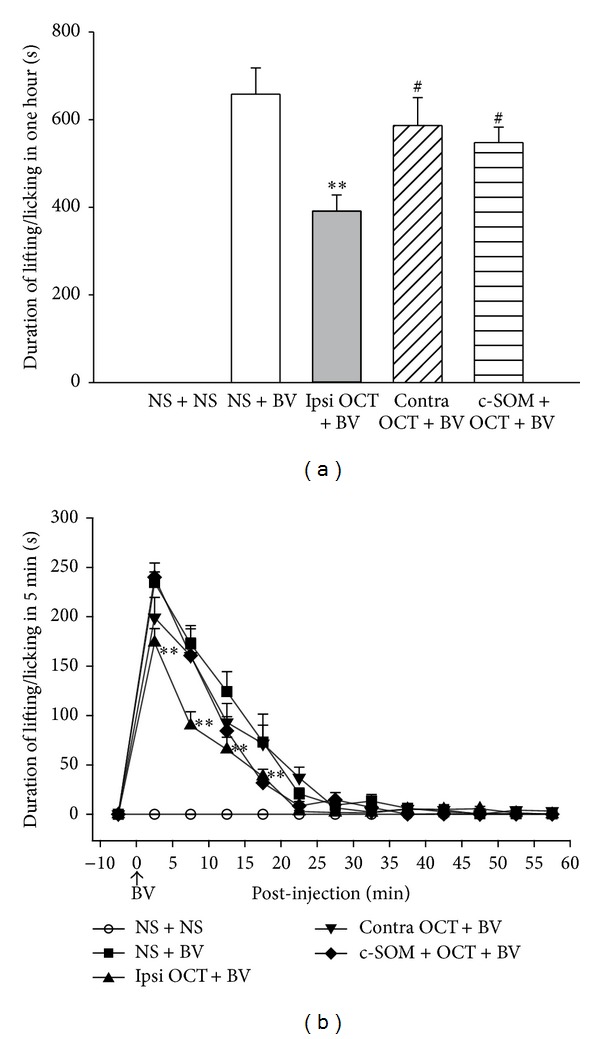
Effects of octreotide on lifting/licking (a) and time course (b). *n* = 18 for the NS + BV and ipsilateral OCT + BV groups, and *n* = 10 for other groups. **P < 0.01, compared with those in the NS + BV group. ^#^ *P* < 0.05, compared with those in the OCT + BV group. One-way ANOVA tests followed by the Student-Newman-Keuls method were used to compare different treatments. NS, normal saline; BV, bee venom; OCT, octreotide; c-SOM, cyclosomatostatin; contra, contralateral; ipsi, ipsilateral.

Compared with the NS + BV group, the cumulative time spent lifting/licking over 1 hour in the ipsilateral OCT + BV group was significantly shorter (391.4 ± 36.9 s versus 658.2 ± 60.0 s, *n* = 18, *P* < 0.05, Figure 1(a)). In addition, there are significant differences between the NS + BV and ipsilateral OCT + BV groups at the 5-, 10-, 15-, and 20-minute time points after injection of BV (Figure 1(b)). To exclude the systemic effect of OCT, the same dose of the drug was subcutaneously preadministered in the contralateral hindpaw of the BV-treated side. Contralateral pretreatment with OCT did not affect BV-evoked nociceptive lifting/licking duration (586.9 ± 63.4 s  *n* = 10, Figure 1(a)). To illustrate the effect of OCT by activation of the somatostatin receptor, the specific antagonist cyclosomatostatin (c-SOM) was applied 10 min before OCT and it reversed the antinociceptive effects of OCT on lifting/licking responses (547.6 ± 35.5 s  *n* = 10, Figure 1(a)).

**Figure 2:**
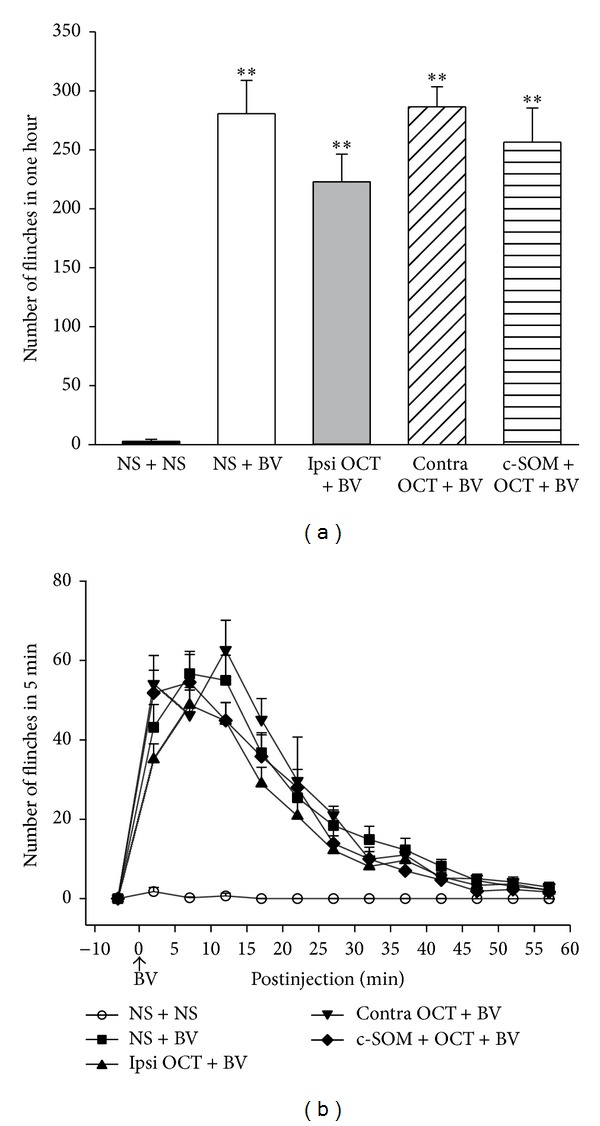
Effects of octreotide on flinch (a) and time course (b). *n* = 18 for the NS + BV and ipsilateral OCT + BV groups, and *n* = 10 for other groups. *P < 0.05, **P < 0.01, compared with the NS + NS group. One-way ANOVA tests followed by the Student-Newman-Keuls method were used to compare different treatments. NS, normal saline; BV, bee venom; OCT, octreotide; c-SOM, cyclo-somatostatin; contra, contralateral; ipsi, ipsilateral.

Similar to other studies [20, 23, 28], intraplantar injection of BV evoked tonic nociceptive flinching behavior with an average of 280.7 ± 28.3 times in 1 hour. Both total flinches in 1 hour and number of flinches at every time point after injection are visually smaller in the ipsilateral OCT + BV group than in the NS + BV group. However, neither cumulative flinches in 1 hour (222.9 ± 23.4 versus 286.4 ± 17.0, *P* > 0.05, Figure 2(a)) nor the number of flinches in each 5 min period was significantly different in the ipsilateral OCT + BV group compared with the NS + BV group (Figures 2(a) and 2(b)).

### 3.2. Effects of Octreotide on BV-Induced Mechanical Allodynia

**Figure 3:**
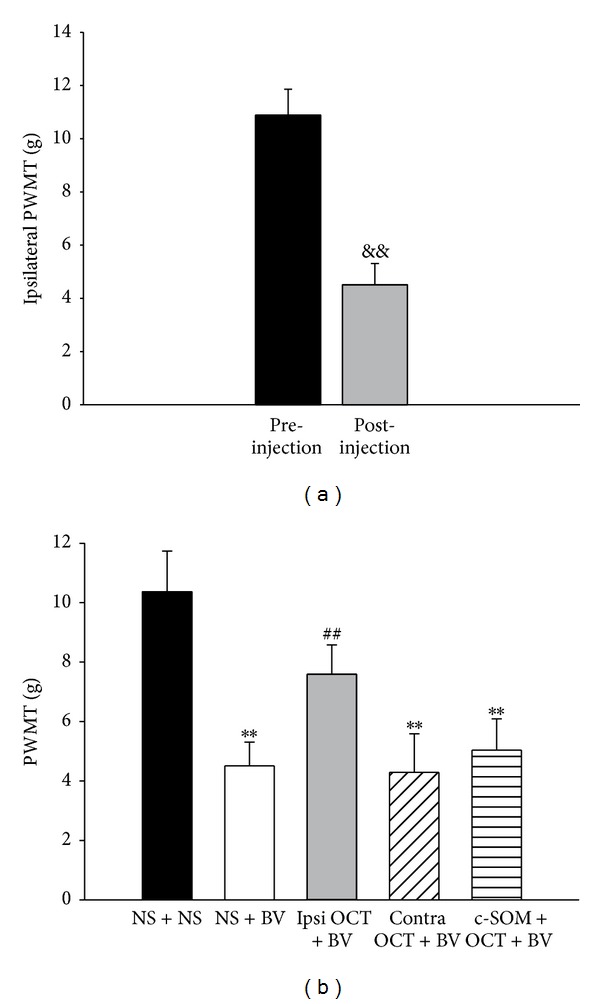
The PWMTs. (a) The ipsilateral pre- and postinjection PWMTs in the NS + BV group. A paired* t*-test was used to compare the PWMTs between pre- and postinjection. (b) The PWMT of the ipsilateral hindpaw after injection of different drugs. One-way ANOVA tests followed by the Student-Newman-Keuls method were used to compare different treatments. *n* = 18 for the NS + BV and ipsilateral OCT + BV groups, and *n* = 10 for the other groups. ^&&^ *P* < 0.01, compared with preinjection in the same hindpaw; **P < 0.01, compared with the NS + NS group; ^##^ *P* < 0.01, compared with the NS + BV group. PWMT, paw withdrawal mechanical threshold; BV, bee venom; OCT, octreotide; ipsi, ipsilateral hindpaw; contra, contralateral hindpaw.

At 2 hours after intraplantar injection of BV, the PWMTs dramatically decreased in the injected hindpaw from 10.89 ± 0.97 g to 4.51 ± 0.80 g, suggesting the occurrence of primary mechanical hyperalgesia (*n* = 18, *P* < 0.01, Figure 3(a)). There was no significant difference between the preinjection and postinjection PWMTs in the contralateral side (11.52 ± 0.87 g versus 11.32 ± 0.94 g). In the NS + NS group, the PWMT did not change significantly after NS injection (ipsilateral: 10.66 ± 1.21 g versus 10.37 ± 1.37 g; contralateral: 10.48 ± 1.12 g versus 10.83 ± 1.40 g; *n* = 10, *P* > 0.05).

The PWMTs in the ipsilateral hindpaw in the NS + BV group were significantly lower after injection than in the NS + NS group (Figure 3(b), *P* < 0.05). However, ipsilateral preapplication of OCT attenuated the PWMTs and there was no significant difference compared with the NS + NS group (*P* > 0.05, Figure 3(b)). This antinociceptive effect on mechanical hyperalgesia did not occur when OCT was injected contralaterally (Figure 3(b)), and intraplantar pretreatment with the somatostatin receptor antagonist c-SOM reversed octreotide's antinociceptive effects (Figure 3(b)).

### 3.3. Effects of Octreotide on Thermal Hyperalgesia Induced by BV

**Figure 4:**
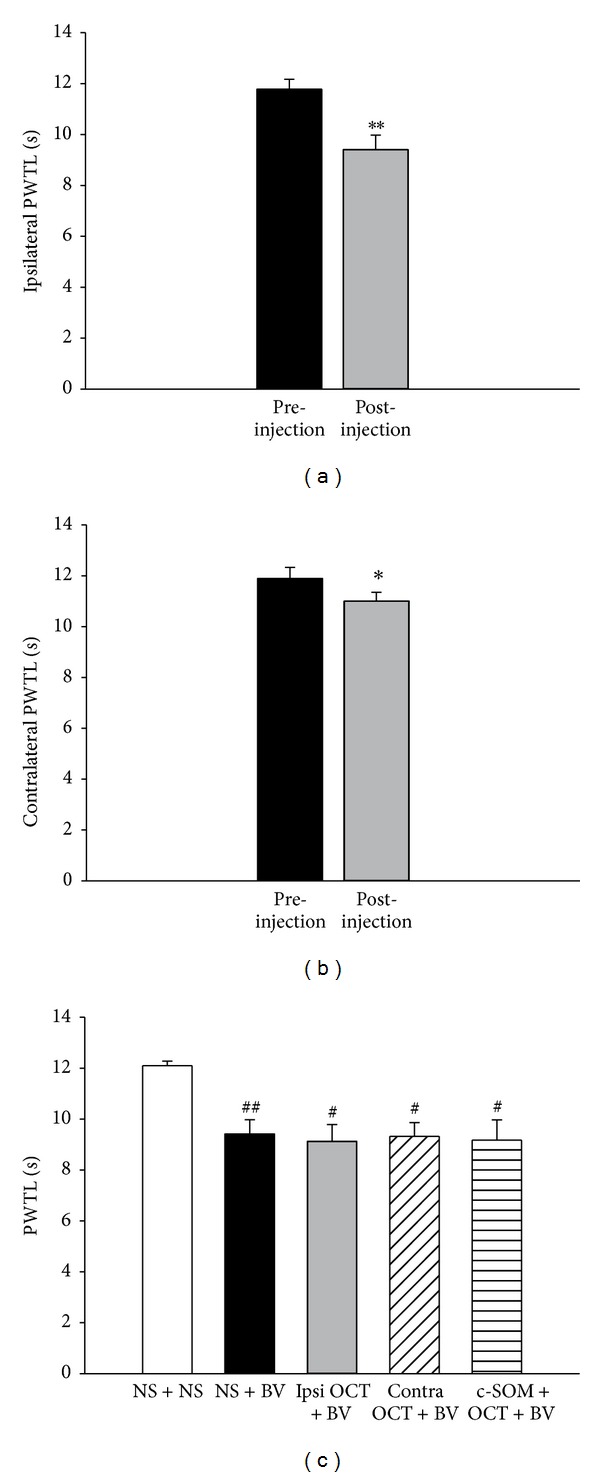
The PWTL. (a) The ipsilateral pre- and postinjection PWTLs in the NS + BV group. A paired* t*-test was used to compare the PWTLs between pre- and postinjection. (b) The PWTL of the contralateral hindpaw in the NS + BV group. A paired* t*-test was used to compare the PWTLs between pre- and postinjection. (c) The ipsilateral postinjection PWTLs of the five different treatment groups. One-way ANOVA tests followed by the Student-Newman-Keuls method were used to compare different treatments. *n* = 18 for NS + BV and ipsilateral OCT + BV groups, *n* = 10 for the other groups. *P < 0.05; **P < 0.01, compared with the preinjection in the same hindpaw; ^#^ *P* < 0.05; ^##^ *P* < 0.01, compared with the ipsilateral postinjection in the NS + NS group. PWTL, paw withdrawal thermal latency; BV, bee venom; OCT, octreotide; ipsi, ipsilateral hindpaw; contra, contralateral hindpaw.

Compared with PWTLs prior to BV injection, the PWTLs in the ipsilateral hindpaw decreased significantly after intraplantar injection of BV in the NS + BV group from 11.78 ± 0.39 s to 9.41 ± 0.57 s (*P* < 0.01, Figure 4(a)), demonstrating primary thermal hyperalgesia. In addition, the PWTLs in the contralateral hindpaw also decreased significantly after injection of BV (11.89 ± 0.44 s versus 11.00 ± 0.35 s; *P* < 0.05, Figure 4(b)), indicating a mirror-thermal hyperalgesia. In the NS + NS group, the PWTL did not change significantly after NS injection.

However, in the ipsilateral OCT + BV group, primary thermal hyperalgesia was still observed (*P* < 0.05). When comparing PWTLs among different groups, PWTLs in the ipsilateral hindpaw after injection of BV in the other four groups were significantly lower than in the NS + NS group (*P* < 0.05, Figure 4(c)). Meanwhile, mirror-thermal hyperalgesia in the ipsilateral OCT + BV group was still observed.

### 3.4. The Expression of pCREB in the Spinal Cord and DRG

**Figure 5:**
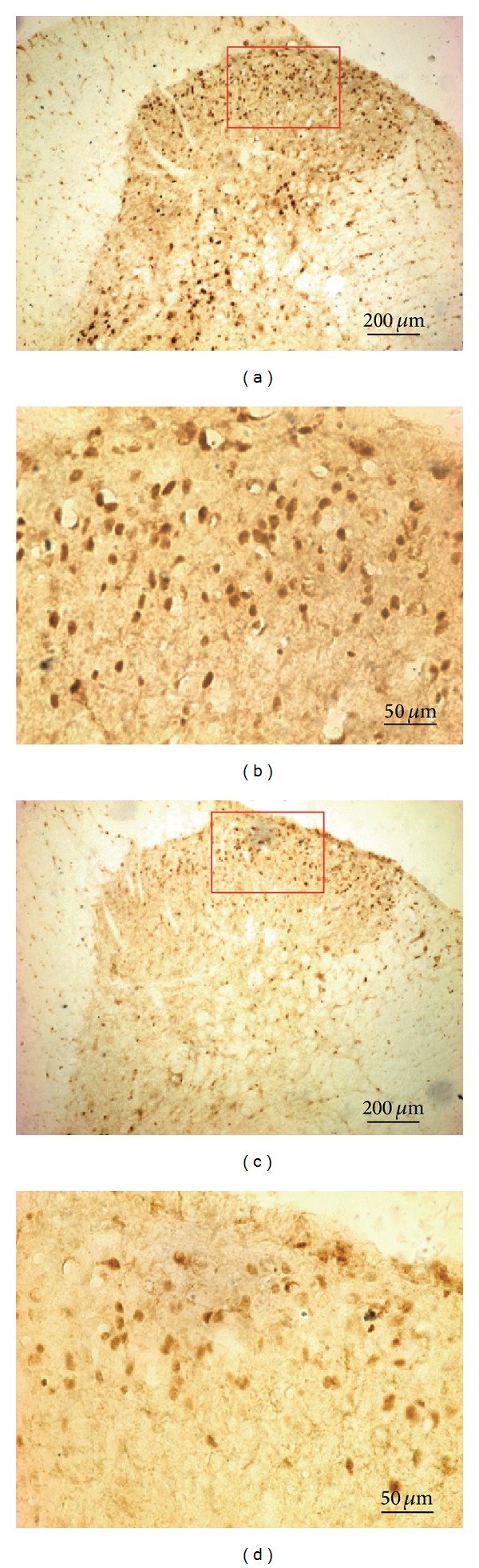
Photomicrograph showing the example of pCREB expression in the L4 spinal cord induced by BV injection following NS or octreotide administration. (a) NS + BV group, (c) OCT + BV group, and (b) and (d) are amplifications of the framed areas in (a) and (c), respectively.

After the injection of BV, the pCREB-positive staining was localized to cell nuclei throughout the bilateral spinal cord and dramatically increased as shown in Table 1 Column 1 and Column 2 (*P* < 0.05). No significant difference was found between the ipsilateral and contralateral sides (*P* > 0.05). In addition, the majority of the pCREB-positive cells were observed in laminae I-II and V-VI in the spinal cord, which is the major terminal of nociceptive afferent fibers and the mediator of nociceptive input. It also showed that preapplication of OCT significantly decreased pCREB expression compared with the NS + BV group (*P* < 0.05), while contralateral pretreatment with OCT and ipsilateral pretreatment with c-SOM + OCT did not reduce the expression of pCREB throughout the spinal cord evoked by BV as shown in Columns 4 and 5.  Figure 5 shows the examples of pCREB expression in the right dorsal horn of the L4 spinal cord.

**Figure 6:**
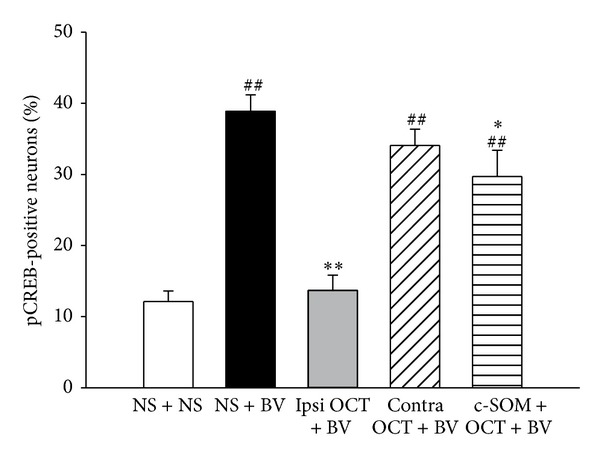
The percentage of pCREB-positive neurons in the ipsilateral DRG in the five groups. One-way ANOVA tests followed by the Student-Newman-Keuls method were used to compare different treatments. ^##^ *P* < 0.01, compared with those in the NS + NS group; *P < 0.05, **P < 0.01, compared with those in the NS + BV group.

**Figure 7:**
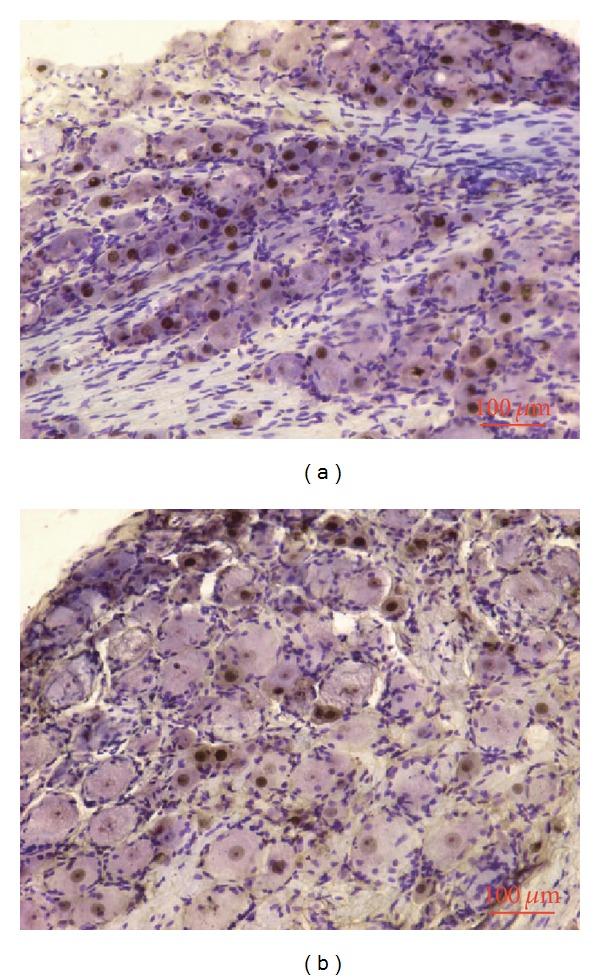
Photomicrograph showing the example of pCREB expression in the L5 DRG induced by BV injection following NS or octreotide administration. (a) and (c) present the example of pCREB expression in the NS + BV group and the ipsilateral OCT + BV group, respectively.

After Nissl restaining of the neuron bodies in the DRG, the expression of pCREB was mostly located in small neurons (Figure 7). Compared with the NS + NS group, the expression of pCREB was significantly increased in the NS + BV group in the ipsilateral DRG (*P* < 0.05, Figure 6). Local injection of octreotide also reduced the BV-induced expression of pCREB in the DRG, and intraplantar pretreatment of the somatostatin receptor antagonist c-SOM reversed the octreotide effects (*P* < 0.05, Figure 6). Figure 7 shows the examples of pCREB expression in L5 DRG neurons.

## 4. Discussion

The most important finding of the present study is that local application of octreotide suppresses the BV-induced nociceptive lifting/licking behavior and pCREB expression in the superficial spinal cord and ipsilateral DRG, and this effect is reversed by pretreatment with cyclosomatostatin, an antagonist of the SSTR.

In the present study, intraplantar injection of BV elicited persistent nociceptive responses such as lifting, licking, and flinches, as well as mechanical allodynia and thermal hyperalgesia, which is consistent with a previous study [20]. BV is a mixed toxin containing melittin, phospholipase A2, apamin, histamine, and mast cell-degranulating peptide [19, 29]. Among these components, melittin is likely to play the most important role in the production of pain, hyperalgesia, allodynia, and inflammatory process [29]. Melittin has been reported to selectively activate capsaicin-sensitive primary afferent fibers [30] and small- to medium-sized DRG cells by opening the nonselective transient receptor potential vanilloid 1 (TRPV1) cation ion channel [31]. Either pre- or posttreatment with capsazepine, a selective blocker of thermal nociceptor TRPV1, significantly prevents or suppresses the persistent, spontaneous nociception induced by intraplantar injection of melittin [29]. These results suggest that the nociceptive and hyperalgesic effects of BV might be mediated by activation of the peripheral capsaicin receptor TRPV1. TRPV1 activation plays a key role in inflammatory pain [4] and TRPV1 knockout mice do not develop inflammatory pain behaviors [32, 33]. TRPV1 is a nonselective cation channel with a preference for calcium [4, 34]. Agonist binding to the TRPV1 operated nonspecific cation channel is likely to induce a conformational change in receptor protein, leading to cation (predominantly calcium) influx [35]. This cation influx may cause membrane depolarization [36]. When membrane depolarization reaches the threshold level, an action potential is generated. The action potential is propagated along the entire length of the neuron and may be perceived as pain by the CNS [34, 37].

The present study showed that intraplantar pretreatment with octreotide attenuated the increase in the duration of lifting/licking induced by BV. However, contralateral octreotide treatment did not demonstrate any antinociceptive effect. Ipsilateral pretreatment with cyclosomatostatin, an antagonist of somatostatin receptors, reversed the effect of octreotide. Thus, the present study indicates that local antinociceptive effects of octreotide occur through the activation of peripheral somatostatin receptors. Five receptor subtypes have been found: SSTR1–5. SSTR1–4 had been identified on afferent terminals and in the DRG [1, 38–40]. In addition, OCT has high affinity to three receptor subtypes (SSTR2, 3, 5) that would be blocked by c-SOM [41]. Moreover, it has been reported that SSTR2 was located in the axon terminal of peripheral unmyelinated nerve fibers [1]. Furthermore, by using SSTR2-deficient mice and immunohistochemistry, the antinociceptive effects of OCT were completely abolished and only SSTR1 and SSTR2A were detected in a subset of small- and medium-diameter neurons in the dorsal root ganglia of naive wild-type mice [42]. Thus, the present study indicates that local antinociceptive effects of octreotide occurred through the activation of peripheral somatostatin receptors, most likely by SSTR2. The results are also consistent with previous studies demonstrating that somatostatin has analgesic effects in rodents and humans by acting on peripheral somatostatin receptors [5, 43, 44]. It is reported that SSTR2 inhibits activity through the presynaptic release of glutamate evoked by TRPV1 in the spinal cord [3]. SST is also synthesized and stored in capsaicin-sensitive transient receptor potential vanilloid 1 (TRPV1) expressing nociceptive afferents, and it has been identified in spinal dorsal horn neurons [45, 46]. Modulation of pain transmission has a complex circuitry that includes SSTR [46, 47]. During the sensitization of nociceptors, it has been demonstrated that SST interacts with the vanilloid receptor TRPV1 [4, 47].

The present study showed that intraplantar pretreatment with OCT depressed BV-induced increases in the duration of L/L but not the numbers of flinches. The mechanism by which flinches and L/L behavior were differentially affected in this study is not clear. The lack of an effect of OCT on flinch behavior suggests that the neural circuit underlying this reflexive behavior presumed to be segmentally organized may be distinct from that underlying L/L behavior, which is more likely to involve supraspinal mechanisms [1, 48].

Another finding in the present study is that intraplantar pretreatment with octreotide inhibits BV-induced mechanical, but not thermal, hyperalgesia. These results are consistent with the previous studies showing that octreotide relieves mechanical hyperalgesia in arthritic DA rats [6] and somatostatin reduces mechanical hyperalgesia in carrageenan-induced inflammation [5]. The inconsistent effect of octreotide on thermal and mechanical hyperalgesia may be due to thermal and mechanical hyperalgesia relying on the activation of two different intracellular cascades of events in the spinal cord. Neural substrates underlying mechanical hypersensitivity and thermal hypersensitivity have been dissociated in a number of ways [49, 50].

Mirror-thermal hyperalgesia was a typical characteristic of the BV test in SD rats that had not been reported in other inflammatory pain models such as nociception induced by formalin [1, 20] and capsaicin [4]. Both NMDA and non-NMDA receptors involved in central sensitization contribute to the development of BV-induced mirror-thermal hyperalgesia [51]. A temporal spinal summation of the ongoing primary afferent input from the BV injection site directly contributed to the development or establishment of the mirror-thermal hyperalgesia. The BV-induced paw flinches may reflect an increase in spontaneous activity of peripheral primary afferents and spinal neurons involved in the BV test [28]. Thus, both peripheral and central mechanisms may be involved in the spontaneous nociceptive behaviors and the thermal hyperalgesia.

The present study demonstrates that BV injection into the rat hindpaw induces, within minutes, the phosphorylation of CREB at the transcriptional regulatory site serine-133 in the spinal cord and ipsilateral DRG neurons. Interestingly, unilateral peripheral inflammation induces this phosphorylation event bilaterally in the spinal cord. These results are similar to the previous reports that pCREB increased after paw inflammation induced by carrageenan and formalin [14, 17], nerve injury [15], and neuropathic pain [16, 17], indicating that pCREB is involved in hyperalgesia and spontaneous nociception. A majority of the cells containing pCREB-positive nuclei in the spinal cord after BV stimulation were observed in laminae I-II and V-VI, which are the regions where a majority of the noxious primary afferents terminate and the cell bodies of nociceptive neurons are localized [52]. Furthermore, pCREB was also observed in a few small DRG neurons, which are involved in mediating pain transmission. pCREB is a component of the intracellular mechanisms active during persistent pain [15, 53]. Taken together, these results indicate that BV-induced expression of pCREB is predominantly confined to the neurons involved in the pain response. CREB binds specifically to the CRE present in the promoters of many genes, including the gene encoding somatostatin, for which transcription rates are strongly regulated by cAMP [54, 55]. Increases in pCREB may be associated with pain-related behaviors. The present study demonstrates that the expression of pCREB in the spinal cord can be suppressed by local application of octreotide, and cyclosomatostatin pretreatment antagonizes this effect. Through activating local SSTR, octreotide decreased the peripheral nociceptive messages transmitted to the spinal cord. Therefore, the nociceptive marker pCREB was reduced in ipsilateral DRG and spinal cord neurons.

In conclusion, the present study showed that octreotide relieved the BV-induced lifting/licking, mechanical allodynia, and spinal pCREB expression, which confirmed that somatostatin exerted peripheral analgesic effects through its receptors. In addition, pCREB may contribute to these nociceptive responses.

## Figures and Tables

**Table 1:** Number of pCREB-positive cells in the ipsilateral and contralateral dorsal horn of the lumbar spinal cord in SD rats.

Subregion	NS + NS	NS + BV	Ipsilateral OCT + BV	Contralateral OCT + ipsilateral BV	c-SOM + OCT + BV
Ipsi I-II	41.06 ± 2.52	127.20 ± 2.96^##^	64.93 ± 4.39^##∗∗^	109.80 ± 6.48^##$$^	110.80 ± 6.65^##$$^
Contra I-II	34.47 ± 3.99	121.40 ± 5.03^##^	63.87 ± 4.09^##∗∗^	112.20 ± 6.09^##$$^	111.20 ± 7.13^##$$^
Ipsi III-IV	5.00 ± 1.41	17.60 ± 1.86^##^	12.20 ± 2.22^##^	15.40 ± 1.53^##^	13.20 ± 2.25^##^
Contra III-IV	4.60 ± 1.06	17.20 ± 1.52^##^	12.40 ± 2.27^##^	15.00 ± 2.28^##^	13.80 ± 1.74^##^
Ipsi V-VI	33.60 ± 5.99	64.80 ± 3.87^#^	38.87 ± 5.95*	54.60 ± 3.12	45.20 ± 8.94
Contra V-VI	25.20 ± 4.11*	61.60 ± 3.19^##^	38.20 ± 8.79*	50.00 ± 3.03^#^	51.00 ± 9.53^#^

Data are presented as the means ± SEM from 5 rats. For each rat, counts from 5 sections were averaged. *P < 0.05; **P < 0.01, compared with the NS + BV group; ^#^ *P* < 0.05; ^##^ *P* < 0.01, compared with the NS + NS group. ^$$^ *P* < 0.01, compared with the OCT + BV group. Ipsi: ipsilateral spinal cord; Contra: contralateral spinal cord.

## Abbreviations

DRG: Dorsal root ganglion
BV: Bee venom
OCT: Octreotide
SD: Sprague-Dawley
PWMT: Paw withdrawal mechanical threshold
PWTL: Paw withdrawal thermal latency
CREB: cAMP response element-binding protein
pCREB: Phosphorylated cAMP response element-binding protein
NS: Normal saline
c-SOM: Cyclosomatostatin
PBS: Phosphate buffer saline
NGS: Normal goat serum
ABC: Avidin-biotin-peroxidase
SST: Somatostatin
SSTR: Somatostatin receptor.
